# Endoscopic rendez-vous after damage control surgery in treatment of retroperitoneal abscess from perforated duodenal diverticulum: a techinal note and literature review

**DOI:** 10.1186/1749-7922-8-26

**Published:** 2013-07-16

**Authors:** Ivan Barillaro, Veronica Grassi, Angelo De Sol, Claudio Renzi, Giovanni Cochetti, Francesco Barillaro, Alessia Corsi, Alban Cacurri, Adolfo Petrina, Lucio Cagini, Carlo Boselli, Roberto Cirocchi, Giuseppe Noya

**Affiliations:** 1 General and Emergency Surgical Clinic. S. Maria Hospital, University of Perugia, Terni, Italy; 2 General and Oncological Surgical Clinic. S. Maria della Misericordia, University of Perugia, Perugia, Italy; 3 Urological Andrological Surgery and Minimally Invasive Techniques. S. Maria Hospital, University of Perugia, Terni, Italy; 4 General Surgical Clinic. S. Maria della Misericordia, Perugia, Italy; 5 Thoracic Surgery Unit. S. Maria della Misericordia, University of Perugia, Perugia, Italy; 6 General and Emergency Surgical Clinic. S. Maria Hospital, University of Perugia, Via Tristano di Joannuccio 1, Terni, Italy

**Keywords:** Duodenum, Diverticula, Complications, Perforation, Surgical treatment

## Abstract

**Introduction:**

The duodenum is the second seat of onset of diverticula after the colon. Duodenal diverticulosis is usually asymptomatic, but duodenal perforation with abscess may occur.

**Case presentation:**

Woman, 83 years old, emergency hospitalised for generalized abdominal pain. On the abdominal tomography in the third portion of the duodenum a herniation and a concomitant full-thickness breach of the visceral wall was detected. The patient underwent emergency surgery. A surgical toilette of abscess was performed passing through the perforated diverticula and the Petzer’s tube drainage was placed in the duodenal lumen; the duodenostomic Petzer was endoscopically removed 4 months after the surgery.

**Discussion:**

A review of medical literature was performed and our treatment has never been described.

**Conclusion:**

For the treatment of perforated duodenal diverticula a sequential two-stage non resective approach is safe and feasible in selected cases.

## Introduction

The duodenum is the most common site for diverticula after the colon [1]. Duodenal diverticula, which can be single or multiple, are found in 5-10% of radiologic and endoscopic exams [2]. In over 70% of cases they are localized in the second portion of the duodenum, less frequently in the third or the fourth one, exceptionally in the first one [2,3]. They are usually asymptomatic; on the other hand they can determine abdominal postprandial pain, dyspeptic disorders or colic-like pains [2]; diverticulitis, bleeding, perforation may rarely occur [4,5]. The first case report of duodenal diverticulosis, describing a diverticulum containing 22 gallstones, was performed in 1710 by Chomel [6]. Surgery is necessary only if symptoms are persistent or if complications arise [7]: the diagnosis of perforated diverticula of the third duodenal portion is late and the management is still matter of debate [8-12]. In this techinal note we report a new sequential treatment of perforated duodenal diverticula.

## Case presentation

**Figure 1:**
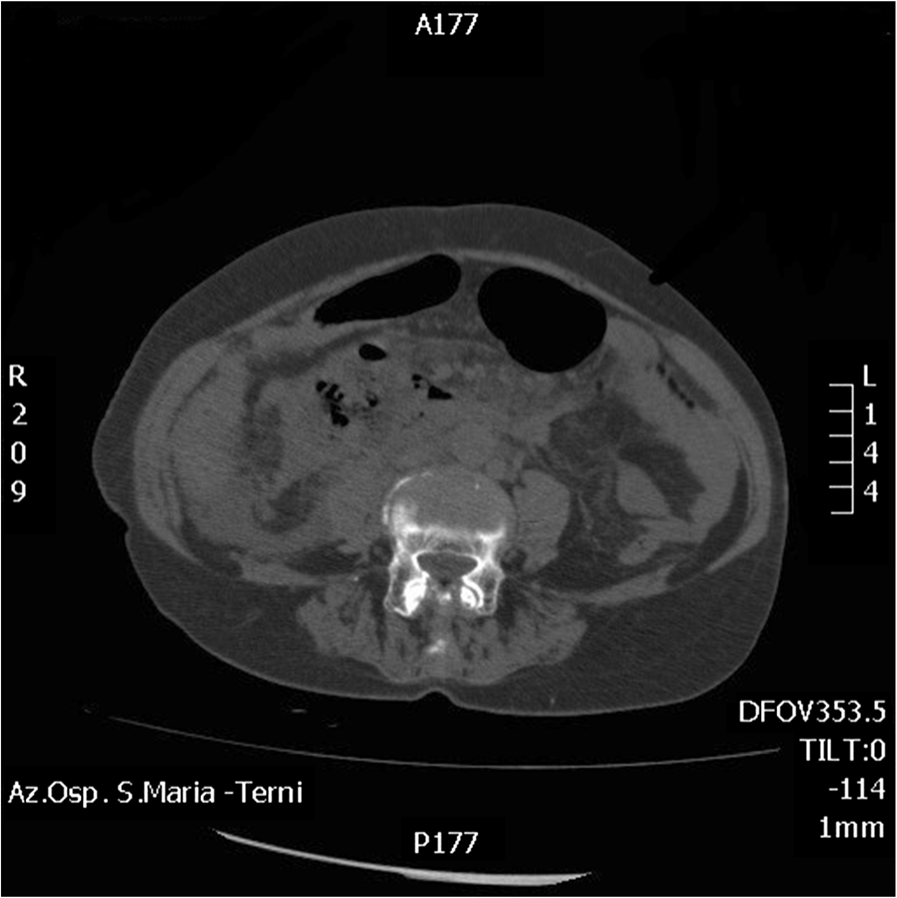
CT on admission.

**Figure 2:**
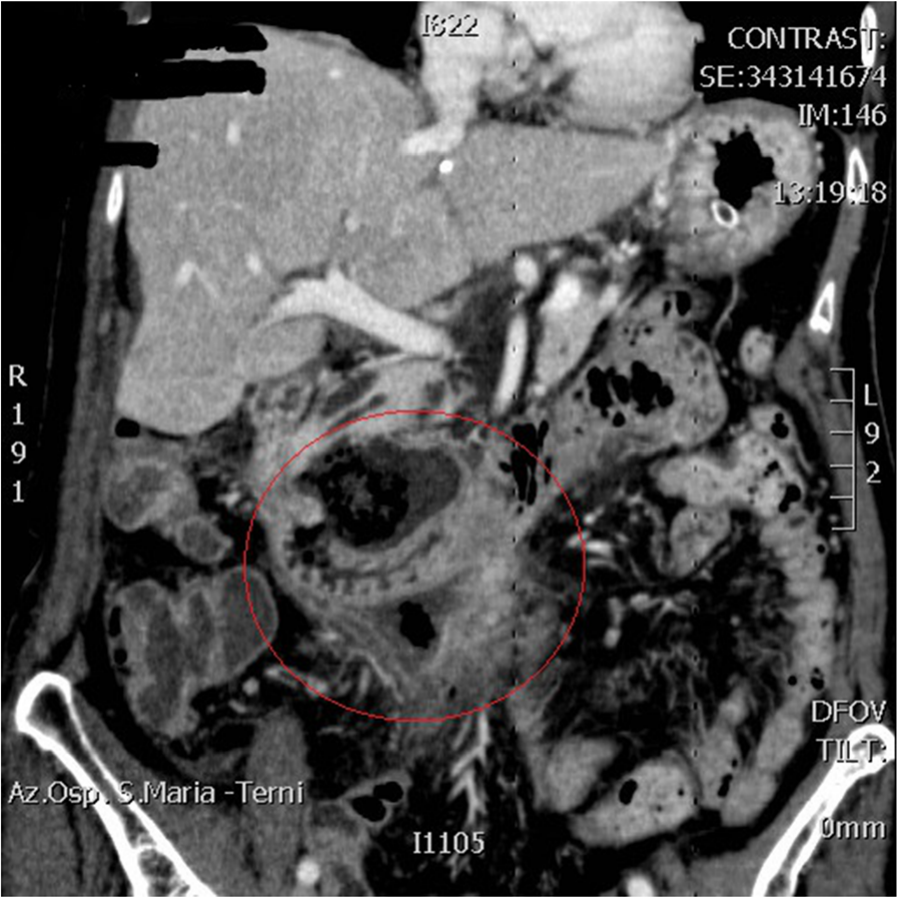
CT after three days from the admission.

**Figure 3:**
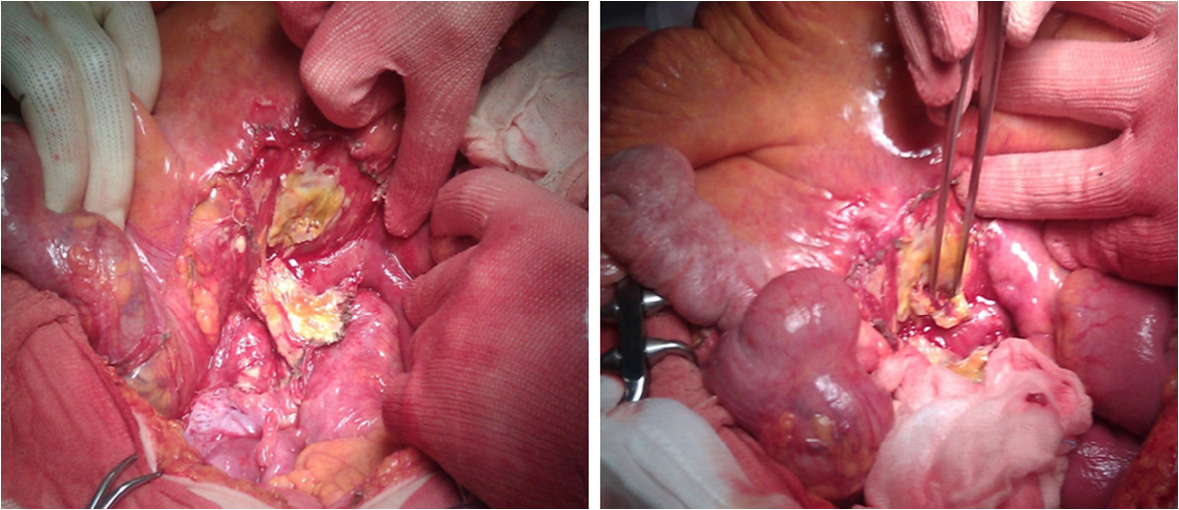
Intraoperative finding.

**Figure 4:**
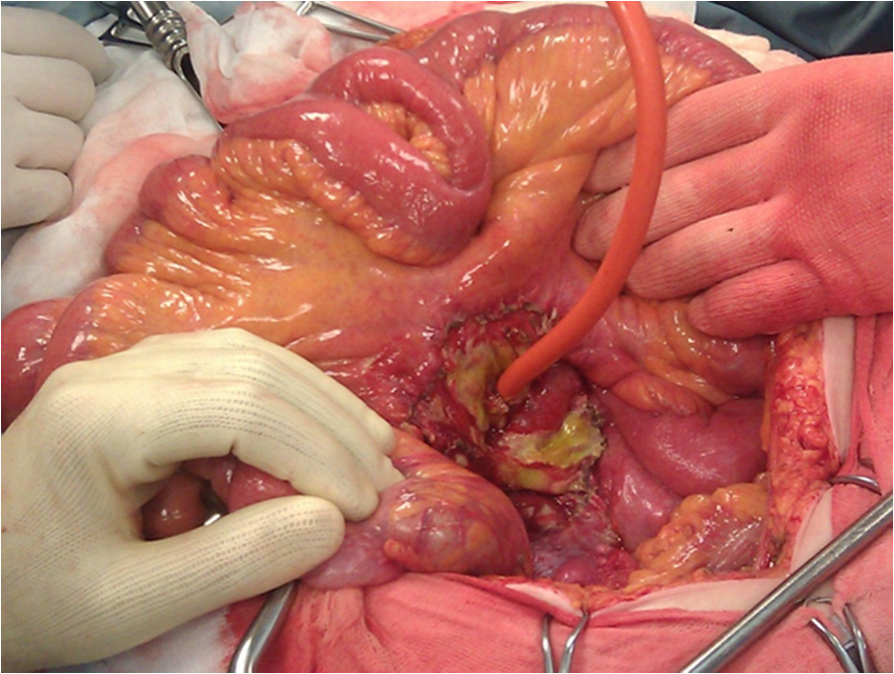
Petzer’s tube drainage placement.

**Figure 5:**
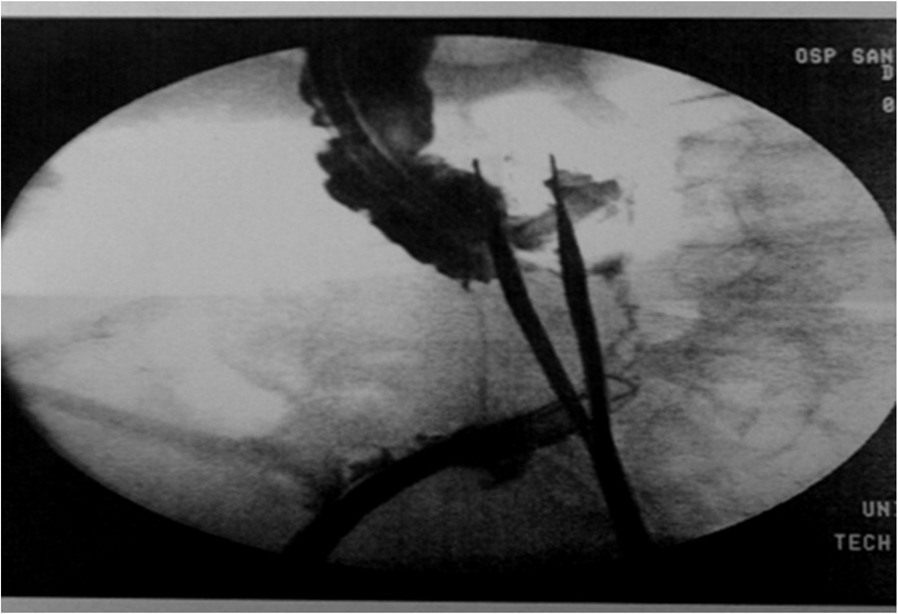
Nasogastric tube positioned in front of the diverticulum, anchored with a thread outside the drainage tube.

Woman, 83 years old, emergency hospitalised for generalized abdominal pain. She reported some alimentary vomiting episodes and diarrheic bowel had occurred during the 3 days before admission and a history of colonic diverticular disease. In the physical examination globular abdomen and pain after deep palpation of the epi-mesogastric region were observed. Laboratory tests resulted within the normal range: leukocytes were 4720/mm^3^ (normal range 4500-10800/mm^3^), hematocrit was 50,5% (normal range 38-46%), haemoglobin was 11.4 g/dl (normal range 12–16 g/dl). The patient underwent plain abdominal X-Ray, which revealed neither free sub-diaphragmatic air nor air-fluid levels. Computed tomography (CT) scans, taken in emergency, showed a densitometric alteration in the periduodenal adipose tissue for the presence of multiple pools which extended along the right lateroconal fascia and occupied the anterior pararenal space, which includes the second and the third portion of the duodenum (Figure 1). At this exam a subtle perihepatic effusion layer was also detected. Within the third day from admission, after the onset of fever, leukocytosis, because of the increase of abdominal pain and the progressive clinical worsening a second abdominal CT scan was performed (Figure 2). This last radiological exam allowed to definitively exclude pneumoperitoneum; a wall herniation in the third portion of the duodenum containing endoluminal material and a breach in the medial wall of the same bowel segment were observed. Furthermore, contiguously to the duodenal breach, within the adipose tissue, in the context of an underlying fluid layer, air bubbles were detected. Being these findings strongly suggestive of a locally confined perforation, the patient in sepsis (temperature 39°C, increased heart rate, leukocytes 16400/mm^3^) underwent emergency surgery. A partial coloepiploic detachment, Kocher manoeuvre to the proximal half of the II duodenal portion and subsequent isolation of the III one were performed; at this level, on the upper edge, a perforated diverticulum occupied the retroperitoneal space and it was partly surrounded by an abscess. The large implant base of the diverticulum prevented both the resection and the direct suture, being the laceration too jagged, thickened and oedematous (Figure 3). The septic condition of the patient prevented a derivation surgery, which would have been time consuming, demolitive and hazardous. A surgical toilette of abscess was performed passing through the perforated diverticula and the Petzer’s tube drainage was placed in the duodenal lumen (Figure 4). On the first post-operative week the patient was fed with parenteral nutrition, on the second week the patient started a liquid diet and on the 15th post-operative day the patient got a solid diet. No postoperative complications occurred and the patient was discharged on the 30th post-operative day. The duodenostomic Petzer was endoscopically removed 4 months after the surgery. The Petzer’s drainage tube was grasped by endoscopic transgastric way and then removed outside by oral way. In relation to the general condition of the patient was necessary to insert a nasogastric tube into the duodenum for 15 days to reduce the possibility of leak. During the procedure a nasogastric tube, previously anchored on the cutaneous edge of the Petzer, was pulled in the duodenum without effort, being the former on guide of the latter. A drainage tube was percutaneously positioned in the fistulous tract with its distal extremity outside the duodenum. Radiologic follow up with Gastrographin® confirmed the right position both of the nasogastric tube in the duodenum at the level of the fistulous orifice and of the drainage tube inside the tract, at about 4 cm from the wall of the duodenum. The drainage tube was left in place for 15 days (Figure 5). This procedure ensures less trauma and fewer potential complications in the subjects strongly debilitated. Fourteen days after, the patient underwent transit X-Ray with Gastromiro® which showed a normal passage of the contrast medium without any sign of spillages or fistulous tracts. Check-up carried out after 12 months shows normal results.

## Discussion

In our techinal note we reported a new surgical treatment of retroperitoneal abscess from diverticular perforation of the III duodenal portion with endoscopic rendez-vous after damage control surgery. The advantage of this technique consists in performing a non-resective approach with no post operative complication rate. Duodenal diverticula located in the first portion have a low incidence; their site is on the anterior face or on the external right curve edge of the duodenum and their surgical management do not present remarkable technical difficulties. Duodenal diverticula are usually asymptomatic, surgery is needed in less than 3% of cases [8], when clinical manifestations or complications are observed. In about 10% of cases duodenal diverticula are symptomatic (bleeding, pain and nausea caused by distension or inflammation) [13,14] and they enter in the differential diagnosis of the acute abdomen [15-17]. Complications of duodenal diverticula are rare, but they could be devasting; the most frequent one is diverticulitis with perforation. Since diverticula of third portion are usually located in the retroperitoneal space, the onset of symptoms is often insidious and diagnosis is often delayed [18]. Even if several cases are described in which a conservative management with antibiotics and percutaneous drainage is preferred [19,20], this treatment should be taken only after a careful consideration. In literature, several types of treatments are described, both surgical or conservative, according to the patient’s condition and the localization of the duodenal diverticulum: segmental duodenectomies, pylorus-preserving pancreaticoduodenectomy (p-p Whipple), diverticulectomies [11]. At the moment, the conventional treatment is diverticulectomy with duodenal closure and drainage positioning, especially when they are located in the retroperitoneal space [21-23]. The revision of the medical literature does not reveal any surgical treatment equal to ours for complicated diverticula in the third duodenal portion. A review of medical literature was performed; the research was restricted to studies published between September 1985 and December 2012. We reviewed 40 studies producing 64 cases. We considered the treatment of the perforated duodenal diverticulum; the results of this review was reported in Table 1. Perforations were most commonly located in the second (78% of cases) and in the third portion of the duodenum (17% of cases). The most common approach is surgical (80% of cases), although only few reports of conservative management with antibiotics and percutaneous drainage are available (3% of cases). The indications to a surgical intervention and eventually the choice of the correct surgical approach, depend on the patient’s clinical situation and intraoperative findings. If the inflammation didn’t severely impair the access to the interested structures and their integrity, the treatment of choice is, after Kocher manoeuvre, diverticulectomy with single or double-layer duodenal closure (45% of cases). It is important to place drainage tubes, especially in the retroperitoneum, if affected. A slice of the greater omentum can be patched over the closure. Injury to the pancreatic or distal common bile duct can be avoided by placing a tube into the ampulla of Vater before dissecting the diverticulum. When there is substantial inflammation of the duodenum, a diversion should be performed by a subtotal gastrectomy followed by Billroth II reconstruction, or a Roux-en-Y gastroenteroanastomosis (12% of cases). Only patients with mild disease are likely to benefit from non-operative management. In the case described above, the demolition of the duodeno-cephalo-pancreatic region, as well as the confectioning of a bilio-digestive anastomosis of hepatic type or a choledochal jejunostomy for bypass purpose, were not affordable because of the septic conditions caused by the purulent peritonitis. Our treatment, to our knowledge, has never been described, and we propose it as a new and innovative treatment for partients whose general conditions do not allow demolitive invasive surgery.

**Table 1:** Kind of treatment of perforated duodenal diverticulum reported in medical literature

**Author**	**Pz**	**Duodenal portion**	**Year**	**Kind of treatment performed**	**Type of treatment**	
					**Surgical**	**Non-surgical**
Thorson CM et al. [11]	4	II portion	2012	Non operative management		Bowel rest antibiotics
Metcalfe MJ et al. [24]	1	II portion	2010	Surgical treatment	Diverticulectomy	
Gottschalk U et al. [25]	1	II portion	2010	Endoscopical treatment		
Lee HH et al. [23]	1	II portion	2010	Surgical treatment	Laparoscopic Diverticulectomy	
Volchok J et al. [26]	1	II portion	2009	Surgical treatment	Diverticulectomy	
Lopez-Zarraga F et al. [27]	1	II portion	2009	Surgical treatment	Diverticulectomy	
Ames JT et al. [28]	8	II portion	2009	Surgical treatment and nonoperative management	NR	Bowel rest antibiotics
		III portion				
Guinier D et al. [29]	1	II portion	2008	Surgical treatment	Diverticulectomy	NR
Schnueriger B et al. [10]	5	II Portion	2008	Surgical treatment and nonoperative management	-Segmental duodenectomy	PTC tube, Bowel rest, Antibiotics
		III Portion				
		IV Portion				
					-Pylorus-preserving duodeno-pancreatectomy (pp-Whipple)	
					-Diverticulectomy	
Martinez-Cecilia D et al. [19]	1	II Portion	2008	Conservative treatment	NR	Bowel Rest, Antibiotics and percutaneous drainage
Huang RY et al. [20]	1	II Portion	2007	Surgical treatment	Diverticulectomy	NR
Hirota S et al. [30]	1	II portion	2007	Surgical treatment	NR	NR
Andromanakos N et al. [31]	1	II Portion	2007	Surgical treatment	Subtotal gastrectomy and antecolic anastomosis and retroperitoneal drainage	NR
Valenzuela Martínez MJ et al. [32]	1	II Portion	2006	Surgical treatment	Diverticulectomy	
Safioleas M et al. [33]	1	II portion	2006	Surgical treatment	Gastrojejunostomy, drenage	
Castellví J et al. [34]	1	III Portion	2006	Surgical treatment	Gastroenteroanastomosis and biliary drainage with Kehr, gastrojejunostomy	NR
Miller G et al. [8]	3	II Portion	2005	Surgical treatment and nonoperative management	Diverticulectomy, diversion (pyloric exclusion, gastrojejunostomy)	Antibiotics, bowel rest
		III Portion				
Papalambros E et al. [35]	1	III Portion	2005	Surgical treatment	Diverticulectomy and duodenostomy at the second duodenal portion	
Lee VT et al. [36]	1	II Portion	2005	Surgical treatment	Roux -en- Y duodenojejunostomy.	
Bergman S et al. [22]	1	II portion	2005	Surgical treatment	Diverticulectomy and duodenotomy	
Marhin WW et al. [37]	2	II portion	2005	Surgical and conservative treatment	Diverticulectomy	Antibiotics therapy
Yokomuro S et al. [7]	1	II portion	2004	Surgical treatment	Primary closure with drainage	
Sakurai Y et al. [6]	1	II portion	2004	Surgical treatment	Diverticulectomy	
Yarze JC et al. [38]	1	II portion	2002	Surgical treatment	Diverticulectomy	
Franzen D et al. [16]	1	II portion	2002	Surgical treatment	Diverticulectomy	
Atmani A et al. [39]	2	II portion	2002	Surgical treatment	Diverticulectomy lateral duodenostomy, T tube	
Gulotta G et al. [40]	1	II portion	2001	Surgical treatment	Diverticulo-jejunostomy on a Roux-en-Y	
Eeckhout G et al. [41]	1	II portion	2000	Percutaneous and endoscopic management		
Tsukamoto T et al. [42]	2	II portion	1999	Surgical treatment and nonoperative management	Diverticulectomy	Antibiotics, percutaneous abscess drainage.
Rao PM et al. [15]	1	III portion	1999	Surgical treatment	NR	
Poostizadeh A et al. [43]	1	III portion	1997	Surgical treatment	Diverticulectomy, Gastrostomy	
Ido K et al. [44]	1	II portion	1997	Surgical treatment	Diverticulectomy	
Cavanagh JE et al. [45]	1	II portion	1996	Surgical treatment	Malecot drainage in diverticulum	
Mehdi A et al. [46]	2	II portion	1994	Surgical treatment	Diverticulectomy	
		III portion				
Guglielmi A et al. [47]	2	II portion	1993	Surgical treatment	Diverticuletomy, diversion	
Pugash RA et al. [48]	2	II portion	1990	Surgical treatment	Aspiration, drainage, T tube	
Steinman E et al. [49]	2	II portion	1989	Surgical treatment	Drainage	
		III portion				
Beech RR et al. [50]	1	II portion	1985	Surgical treatment	Tube duodenostomy	
Stebbings WS et al. [51]	2	I portion	1985	Surgical treatment	Diverticuletomy, primary closure with drainage	

## Conclusion

Our two-stage technique consisting in damage control surgery and endoscopic review enabled us to treat a patient with retroperitoneal abscess from the third portion of the duodenum for which a more demolishing surgical procedure was not recommended. This method implies a close multidisciplinary relation between the surgeon, the endoscopist and the interventional radiologist.

## Consent

Written informed consent was obtained from the patient for publication of this Case report and any accompanying images. A copy of the written consent is available for review by the Editor-in-Chief of this journal.

## Abbreviations

CT: Computed tomography.

## Competing interests

The authors declare that they have no competing interests.

## Authors’ contributions

RC, AD, IB, AC were involved in pre-operative diagnosis and postoperative care. RC and CB conceived the study and participated in the design of the study. IB and VG wrote the manuscript. CR and FB participated in preparation of the figures. AC, LC, AP, GC helped in literature research and critically revised the manuscript. RC and GN coordinated the study. All authors contributed and approved the final version of the manuscript.
